# Metaproteomics analysis of microbial diversity of human saliva and tongue dorsum in young healthy individuals

**DOI:** 10.1080/20002297.2019.1654786

**Published:** 2019-08-26

**Authors:** Alexander Rabe, Manuela Gesell Salazar, Stephan Michalik, Stephan Fuchs, Alexander Welk, Thomas Kocher, Uwe Völker

**Affiliations:** a Interfaculty Institute for Genetics and Functional Genomics, Department of Functional Genomics, University Medicine Greifswald, Greifswald, Germany; b Department of Infectious Diseases, Division of Nosocomial Pathogens and Antibiotic Resistances, Robert Koch-Institute, Wernigerode, Germany; c Department of Restorative Dentistry, Periodontology, Endodontology, and Preventive and Pediatric Dentistry, University Medicine Greifswald, Greifswald, Germany

**Keywords:** Saliva, tongue, metaproteomics, healthy human oral microbiome, nLC-MS/MS

## Abstract

**Background**: The human oral microbiome influences initiation or progression of diseases like caries or periodontitis. Metaproteomics approaches enable the simultaneous investigation of microbial and host proteins and their interactions to improve understanding of oral diseases.

**Objective**: In this study, we provide a detailed metaproteomics perspective of the composition of salivary and tongue microbial communities of young healthy subjects.

**Design**: Stimulated saliva and tongue samples were collected from 24 healthy volunteers, subjected to shotgun nLC-MS/MS and analyzed by the Trans-Proteomic Pipeline and the Prophane tool.

**Results**: 3,969 bacterial and 1,857 human proteins could be identified from saliva and tongue, respectively. In total, 1,971 bacterial metaproteins and 1,154 human proteins were shared in both sample types. Twice the amount of bacterial metaproteins were uniquely identified for the tongue dorsum compared to saliva. Overall, 107 bacterial genera of seven phyla formed the microbiome. Comparative analysis identified significant functional differences between the microbial biofilm on the tongue and the microbiome of saliva.

**Conclusion**: Even if the microbial communities of saliva and tongue dorsum showed a strong similarity based on identified protein functions and deduced bacterial composition, certain specific characteristics were observed. Both microbiomes exhibit a great diversity with seven genera being most abundant.

## Introduction

The human oral cavity with its various hard (teeth with supragingival and subgingival plaque) and soft tissues (tongue, throat, tonsils, cheeks) forms a complex ecosystem for more than 700 different species and phylotypes [1–6]. Current estimations indicate that saliva and dental plaque contain up to 10^9^ and 10^11^ bacteria per ml [7], respectively. Thus, the oral cavity is the second largest microbial ecosystem in humans after the intestine [4,8].

Saliva is the most interesting biofluid in the oral cavity, as it comes into contact with all surfaces and thus represents a fingerprint of the general composition of the oral microbiome [5]. However, other microenvironments also need to be investigated in a comparative way to obtain a comprehensive view of the oral microbiome [9–11]. Therefore, it is not surprising that the analysis of the tongue microbiome is also gaining more and more attention, since tongue diagnostics has been used in traditional Chinese medicine since more than 3,000 years to assess the patient’s state of health [12,13].

Traditional knowledge and current scientific studies have shown that a shift in the balance of the oral bacterial composition can indicate pathological changes [13]. This includes diseases such as halitosis [14,15], dental caries [16] and periodontitis [17,18] as well as systemic diseases like diabetes [19], respiratory diseases [20], cardiovascular diseases [21] and even cancer [9,22] due to the production of pro-inflammatory mediators [23].

However, initially the healthy microbiome [1,24–27] has to be defined before disease-related or disease-causing alterations can be described, which might ultimately lead to the development of diagnostic tools for better treatment or prevention of disease [2,28,29]. Many studies have already been initiated for this purpose using next generation sequencing [9,25,30–34]. Metagenomics provides an impression of the diversity of organisms on the tongue but also of the metabolic potential which might be present [29,35]. As a complementary approach, metaproteomics offers a possibility to measure protein intensities to capture active protein functions and taxonomic units within the microbiome. Furthermore, simultaneous analysis of the microbial and human proteome can also provide insights into interactions between microbes and their host [35].

For our community proteomics study, we collected saliva and tongue samples from 24 oral healthy volunteers [36]. Our primary goal was to describe and compare the microbial composition of saliva and tongue dorsum based on metaproteome data of young healthy individuals. We combined different open-source software applications, which were mainly developed for the analysis of metaproteome data. At the same time, we compared our pipeline to other salivary metaproteomic studies [37–40] to gain information on effectiveness and accuracy.

## Material and methods

### Study population

Saliva and tongue samples were collected from 9 male and 15 female dental students from the dental school of the University Medicine Greifswald. The range of ages was 20–30 years with an average age of 25 years. They were non-smokers, no alcohol or drug addicts and had no systemic disease or antibiotic treatment within the last six months. Further, the subjects were not taking medication permanently. Women during pregnancy or breastfeeding were not considered. The oral health of the volunteers was ensured by the fact that the students examined each other under the guidance of an experienced dentist fulfilling the inclusion criteria: no cavitated teeth, maximal two fillings, probing depth (≤ 3 mm) and bleeding on probing value of less than 10%. The subjects included did not eat, drink or brush their teeth during 5 h before sampling, which was done during the students’ university course in the late morning and early afternoon. The ethics council of the University Medicine of Greifswald approved our study and it was carried out in compliance with the recommendations of the Helsinki Declaration as amended by Somerset West in 1996.

### Sampling

#### Saliva

Stimulated saliva was collected with a commercially available paraffin chewing gum (Ivoclar Vivadent GmbH, Ellwangen, Germany) based upon a modified protocol published previously [41]. Volunteers chewed the paraffin gum for 1 min to stimulate natural salivation. During the chewing process, the subjects collected saliva in the oral cavity and spat into a sterile 50 ml Falcon tube for several times. Twenty µl of a protease inhibitor (Sigma Aldrich, St. Louis, MO,; v/v 1:20) per 1 ml collected saliva was added to prevent protein degradation by proteases. For transportation the collected saliva was stored on dry ice and finally at −80°C until use [36].

#### Tongue samples

Tongue samples were taken from the middle third of the outstretched tongue dorsum with a sterile wooden spatula (NOBA Verbandmittel Danz GmbH and Co KG, Wetter, Germany), 18 mm x 150 mm. The sterile wooden spatula was pressed onto the tongue for 5 swith light and even pressure and then turned over to repeat the process on the other side. After this procedure, the spatula was transferred into a 50 ml Falcon tube containing 2 ml sterile 1 x PBS (gibco®, Thermo Fisher Scientific, Waltham, MA; pH = 7.4) and 40 µl of a protease inhibitor and vortexed for 30 s. The spatula was discarded. For transportation the sample was stored on dry ice and then stored at −80°C until further processing.

### Sample preparation

#### Cell disruption

Saliva preparation were performed using a published protocol [40], which was slightly modified [36]. The collected saliva was first thawed on ice and centrifuged for 15 min at 4°C at 11,500 g. The supernatant was discarded, and the remaining pellet was resuspended in 700 µl TE buffer (10 mM Tris; 1 mM EDTA; pH 8.0). An ultrasound treatment (Labsonic U – B. Braun Melsungen AG, Melsungen, Germany) was carried out for 3 × 30 s on ice (50% power of the device) to disrupt the cells in the pellet followed by another centrifugation step (30 min, 4°C, 16,200 g). The supernatant was stored on ice for further preparation.

For the tongue samples, our preliminary tests showed that the prior vortexing of the sample in connection with the Freeze-and-Thaw process in sterile 1 x PBS (gibco ®, pH 7.4 – CaCl_2_ – MgCl_2_) is a well-suited cell disruption method for this sample type.

#### MS sample preparation

After thawing on ice, 1 ml of the respective supernatant of the tongue samples and 700 µl of the prepared saliva were used for protein precipitation by TCA. DTT was added to the samples (0.02 g/100 µl), samples were vortexed for 10 s and incubated at 37°C for 30 min. For the subsequent precipitation of the proteins, TCA (100%) was added up to a final concentration of 15% and samples were stored on ice for 60 min. The precipitated samples were centrifuged for 45 min (17,000 g, 4°C). To remove the TCA, supernatants were discarded, 500 µl of 100% cold acetone was added and centrifuged for another 15 min (17,000 g, 4°C). The washing step was repeated once again. Samples were vacuum dried for 1 min. The remaining pellets were diluted in 50 µl (saliva) and 35 µl (tongue) 1 x UT solution (8 M urea/2 M thiourea). To define the technical variance and the reproducibility of our study, all samples were prepared in triplicates. A Bradford assay [42] was performed to determine the protein concentration of saliva (Ø 6.4 µg/µl ± 2.3 µg/µl) and tongue samples (Ø 1.7 µg/µl ± 1.6 µg/µl). Four µg protein were reduced with DTT (2.5 mM final concentration, incubation for 60 min at 60°C) and alkylated with IAA (10 mM final concentration, incubation for 30 min at 37°C in the dark). After a 1:10 dilution of the 1 x UT solutions, protein digestion was conducted with trypsin in a ratio of 25:1 (w/w) over a period of 17 h. Peptide mixtures were purified with ZipTipC_18_ material.

### NLC MS/MS measurement

**Figure 1.**
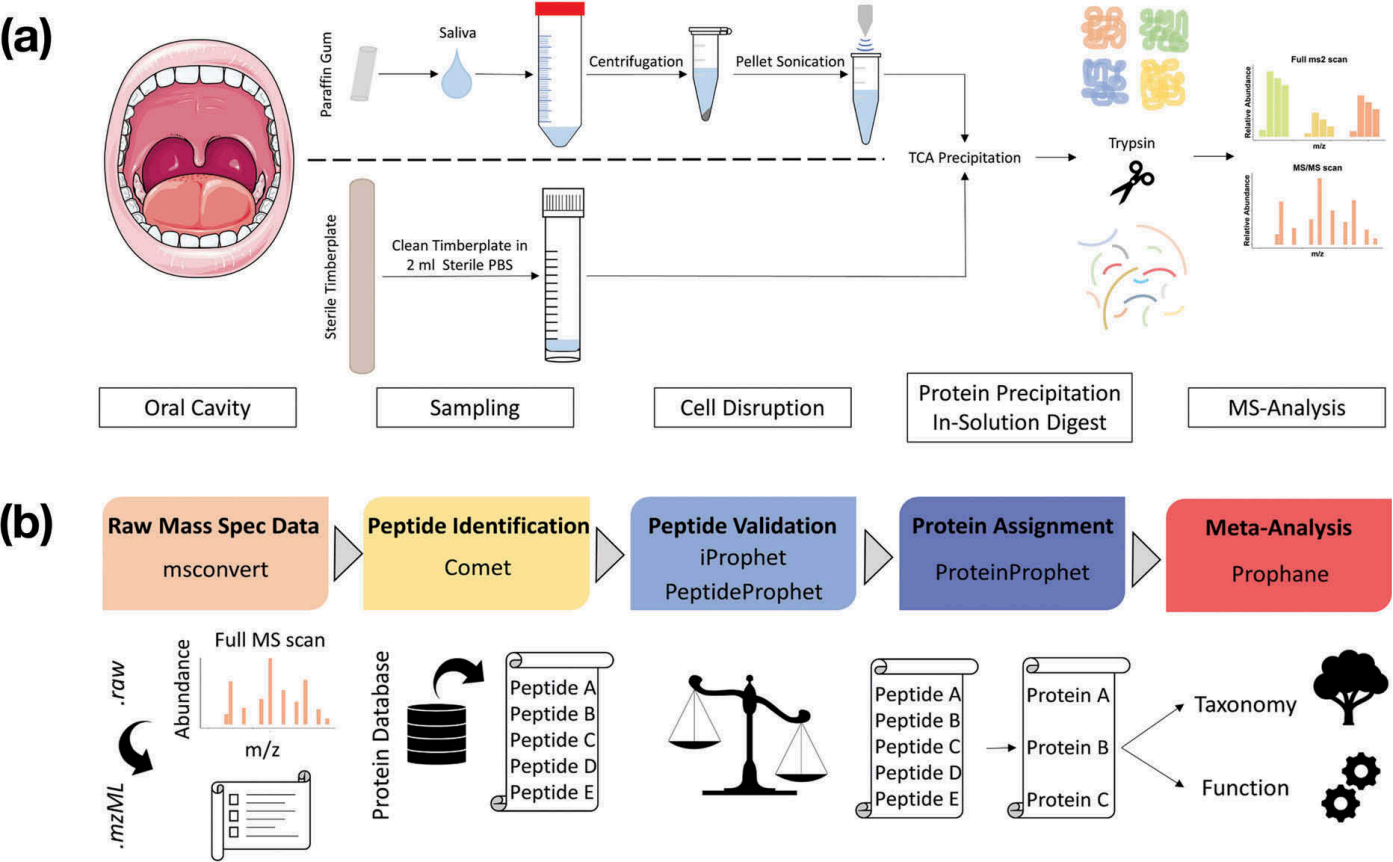
Laboratory workflow for saliva and tongue microbiome analysis (a). Tongue samples were collected with a sterile wooden spatula and transferred into sterile PBS. Salivation was stimulated by chewing a paraffin gum and the subjects spit into a Falcon tube®. Saliva was centrifuged and the resulting pellet was solved in TE-buffer and treated with ultrasonication. Proteins from saliva and tongue samples were precipitated with TCA and digested with trypsin. Peptide mixtures were measured with a Q Exactive™ Plus (LC-MS/MS). Bioinformatic workflow for metaproteomic data analysis (b). The Trans-Proteomic Pipeline was used for the following four steps: (1) Raw-data conversion to mzML-data format. (2) MS/MS database search by the Comet project for peptide identification based on a combined database (Human Swissprot + Human Oral Microbiome Database). (3) Validation of identified peptides. (4) Protein assignment and data filtering by stabilizing false discovery rates (mFDR, pepFDR) with a protFDR of 5.0 %. Finally, the online web-tool Prophane was applied to conduct taxonomic and functional prediction and the statistical analyses were performed in R.

Proteolytic digestion of the proteins with trypsin was followed by analyzing the 144 samples using nano-LC-MS/MS (Supplemental Table 1). The complex peptide mixtures were separated according to their physicochemical properties by means of a reverse phase nano HPLC on an Ultimate® 3000 Nano HPLC (Thermo Scientific). The peptide mixtures were loaded onto a precolumn (Acclaim PepMap100, Thermo Scientific: 75 µm inner diameter, 3 µm C_18_-particles), subsequent separation of the tryptic peptides took place on a 25 µm analytical column (Accucore PepMap RSLC, Thermo Scientific: 25 cm x 75 µm, 2.6 µm C18 particles) via a linear gradient (120 min, 2–25% buffer B) using a binary buffer system consisting of 2% acetonitrile in 0.1% acetic acid (buffer B). The mass spectrometric data were acquired by means of a data-dependent acquisition procedure on a QExactive™ Plus as described before [36] and revealed 5,749,982 spectra. To assure a high quality of our MS data, only spectra with a mFDR ≤ 0.06% were accepted, resulting in 1,933,390 spectra. A complete overview of the laboratory workflow is given in Figure 1(a). All metaproteomic data sets were uploaded to the publicly accessible *MassIVE* database with the dataset link ftp://massive.ucsd.edu/MSV000084137 and doi:10.25345/C53H2C.

### Data analyses

To evaluate our metaproteomic data we used a two-step data analyzing pipeline (Figure 1(b)). We identified spectra, peptides and proteins with stringent criteria using the *Trans-Proteomic Pipeline* (http://tools.proteomecenter.org/software.php), which was developed at the Seattle Proteome Centre. The annotation of the proteins regarding their taxonomy and cellular function was performed by Prophane (www.prophane.de), developed at the Institute for Microbiology at the University of Greifswald.

In detail, *msconvert* (version: 3.0; peak picking based on Vendor algorithm – MS Levels: 1–2) converted the .*raw*-files into readable .*mzML*-format [43]. Peptides were identified using the *Comet* algorithm (http://comet-ms.sourceforge.net/, version: 2016.01 rev. 2) [44,45]. The sequence database (size: 964 MB) consisted of 1,079,744 sequences from the human oral microbiome database (HOMD, www.homd.org, 12/08/2016) [1,46], 20,154 human sequences from UniProt (UniProtKB/Swissprot, www.uniprot.org, 01/06/2016) [47] and their reverse sequences for decoy searches to calculate the false discovery rate. The search algorithm considered trypsinated proteins with a maximum of two missed cleavages. Peptide masses were not allowed to exceed the tolerance range of ± 10 ppm and only monoisotopic masses were included into the analyses. Variable oxidations of methionine [+15.9949] and fixed carbamidomethylation of cysteine [+57.021464] were also considered. Peptides identified by *Comet* [44,45] were weighted and the probability for their existence was calculated with the modules *iProphet* [48], *Peptide Prophet* and filtered using Mayu (version: 1.08) [49]. The *ProteinProphet* assigned the peptides to their corresponding proteins and were accepted with a false discovery rate < 0.05. All proteins, which were covered with at least one unique peptide, were extracted from the data set by an R script (version: 3.4.1) [50] and finally uploaded into the tool Prophane (www.prophane.de). To determine the taxonomic origin of the proteins, Prophane used the Lowest-Common-Ancestor algorithm [51] based on the results of BLASTP (e-value: < 0.01) [52,53] and the database described above. Proteins of bacterial origin are referred as metaproteins, because proteins of one protein group can be assigned to one or more species [54,55]. Thereby, the term ‘meta’ indicates that a different taxonomic distribution could form the basis of a protein group [56]. Metaproteins are referred to as ‘heterogeneous’, if an assignment was not successful on the corresponding taxonomic level (www.prophane.de). (Meta-)Protein functions were classified according to COG/KOG classification (RPS-BLAST 2.2.28+ algorithm; e-value: < 0.01) [57]. The relative quantification of the proteins was performed by spectral counting [58]. The MS/MS spectra obtained were counted and then normalized by prophane using the normalized spectral abundance factor (NSAF-values) [59–62].

### Statistical analyses

The evaluation and statistical analyses were performed in R (version 3.4.1) [50]. In general, a global median normalization was performed for the raw NSAF values. The mean value was calculated for the three measured replicates per sample. Depending on the respective analysis, the sums of the mean NSAF values were calculated to sum up subject-specific spectra per metaprotein, protein, genus or functional subrole.

The factomineR package (version: 1.36) [63] was used for PCA analyses. We did not include missing values and subtracted the column means from their corresponding columns. The centered columns were divided by their standard deviation to unify variance scaling of the data. The data were log_2_ transformed.

We used the metacoder package (version: 0.1.3) to create heat trees for taxonomic analyses [64]. For Figure 3 A/B, the sum of the log_2_ transformed column means (color intensity) was plotted against the sum of the spectral counts (thickness of the individual branches) per taxonomy. For Figure 5, the ratios of the column mean between saliva and tongue were calculated and plotted against the sum of the spectral counts. Resulting missing values were removed.

Our statistical analyses were based on a paired two-sided Wilcoxon signed rank test. The confidence interval was set at 0.95 and the p-value was adjusted for multiple testing using the Benjamini-Hochberg method. A fold-change cutoff = 1.5 and a p-value cutoff = 0.05 were set for the volcano plots.

## Results

### General metaproteome data

We collected one saliva and one tongue sample from each of the 24 subjects and prepared them in three technical replicates. Based on our quality criteria and the combined database of human and bacterial protein sequences, 31,386 distinct peptides for saliva and 31,215 distinct peptides for tongue samples were identified (pepFDR ≤ 1.43%) and assigned to proteins (Supplemental Table 2).

To decrease the number of shared peptides and thus the likelihood of incorrect assignments, only proteins containing at least one unique peptide and a protFDR ≤ 5.0 % were considered resulting in 4,280 saliva proteins of which 1,647 proteins were of human origin and 2,633 bacterial metaproteins. In tongue samples 4,644 proteins were identified of which 1,337 were human proteins and 3,307 bacterial metaproteins.

**Figure 2.**
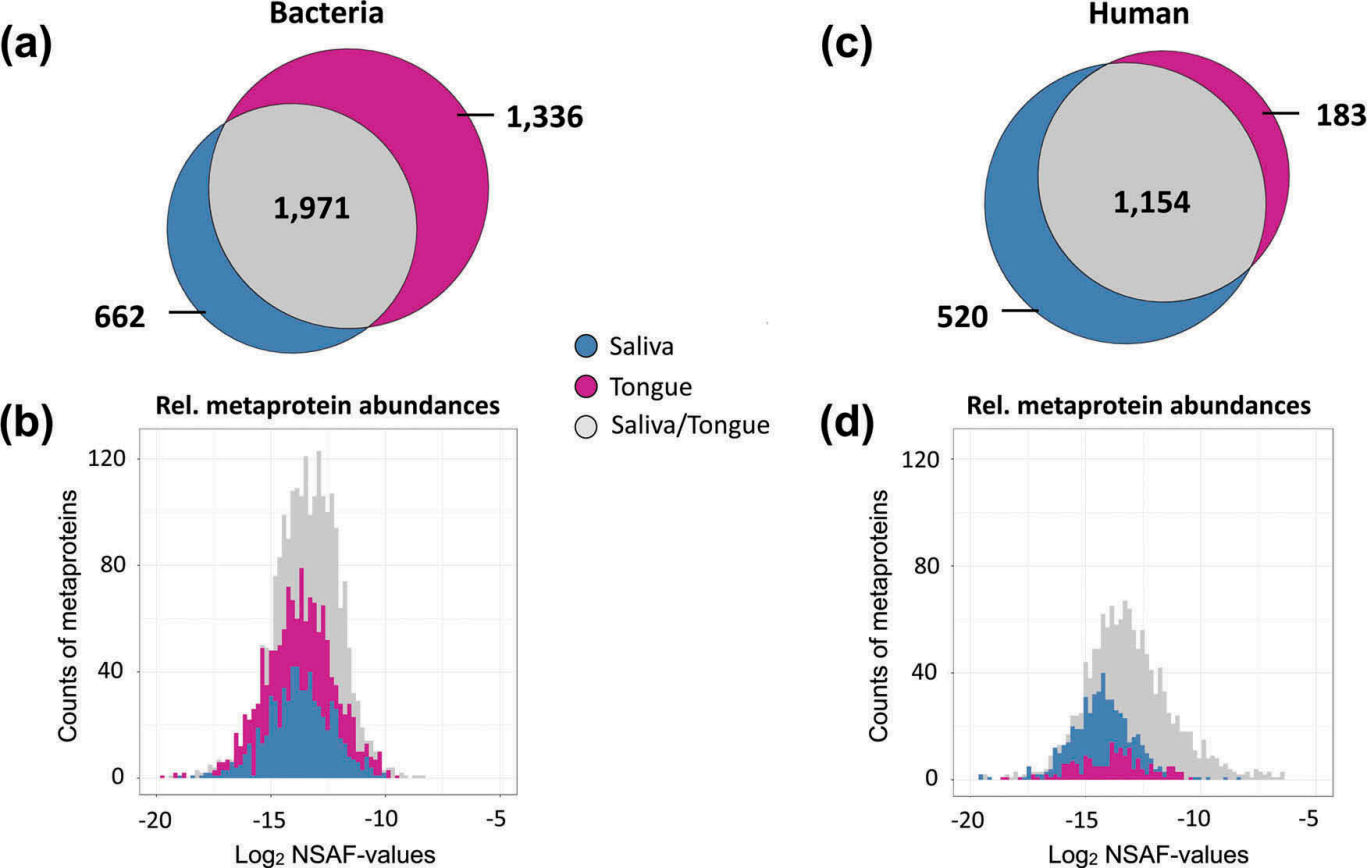
Venn diagrams displaying the number of identified metaproteins in the studied saliva and tongue samples for bacteria (a) and human species (c). Histograms of relative metaprotein abundances based on log_2_ normalized spectral abundance factors (NSAF-values) [60] for bacterial (b) and human proteins (d). The figure emphasizes the distribution of metaproteins for saliva (blue), tongue (red) or shared between both (grey).

To quantify our identified proteins, we used a relative quantification approach. For this purpose, Prophane was used to calculate the normalized spectral abundance factor (NSAF-values) based on spectral counts [60] using the longest sequence in each protein group. Our data showed variations regarding the proportions of human and bacterial abundances in our samples. While human proteins accounted for 78.2% and bacterial metaproteins for 21.7% in saliva, the ratio was different for the tongue. Human proteins accounted for only 59.1% whereas the proportion for bacteria was almost twice as high (40.8%). These differences were also reflected regarding the number of identified proteins. At least 50% of the bacterial and human proteins (Bacteria: 1,971 metaproteins and Humans: 1,154 proteins) could be identified in both the saliva and on the tongue (Figure 2(a,c)). However, more than twice as many specific bacterial metaproteins could be identified for the tongue (1,336 metaproteins) compared to saliva (662 metaproteins), which was also associated with the above mentioned higher relative abundance (Figure 2(a,b)). For the human salivary proteins, we observed the opposite. 520 proteins were only found in saliva, almost three times as many specific proteins (Figure 2(c,d)) as on the tongue (183 proteins).

A principal component analysis (PCA), which was performed with the relative protein intensity data revealed that the interindividual variance was by far greater than the technical variance (Supplemental Figure 1 and 2). We assessed the inter-subject variability (biological CV) and nLC-MS/MS measurement accuracy between the triplicates for each saliva and tongue sample (technical CV) based on the calculation of the coefficient of variance on NSAF values [60]. Our calculations showed an averaged biological CV of 32% for saliva and tongue. The technical CV for those samples was clearly lower for the tongue (18%) and the saliva (16%) samples.

Furthermore, we were interested in the degree of increase for protein identification by measuring the samples in triplicates. We found that the identification rate of proteins increased after two measurements by an average of 17.3% and after the third measurement by additionally 9.3%. Thus, including the results of three technical replicates increased the number of covered proteins by 28.2%.

### Taxonomic profile of saliva and tongue

The taxonomic composition of the oral microbiome has been shown to have an impact on human health [8]. For each protein the taxonomic assignment of the best hit of BLAST [52,53] against the NCBI nr database was used to get a first impression of the diversity and quantity of bacteria in saliva and on the tongue.

**Figure 3.**
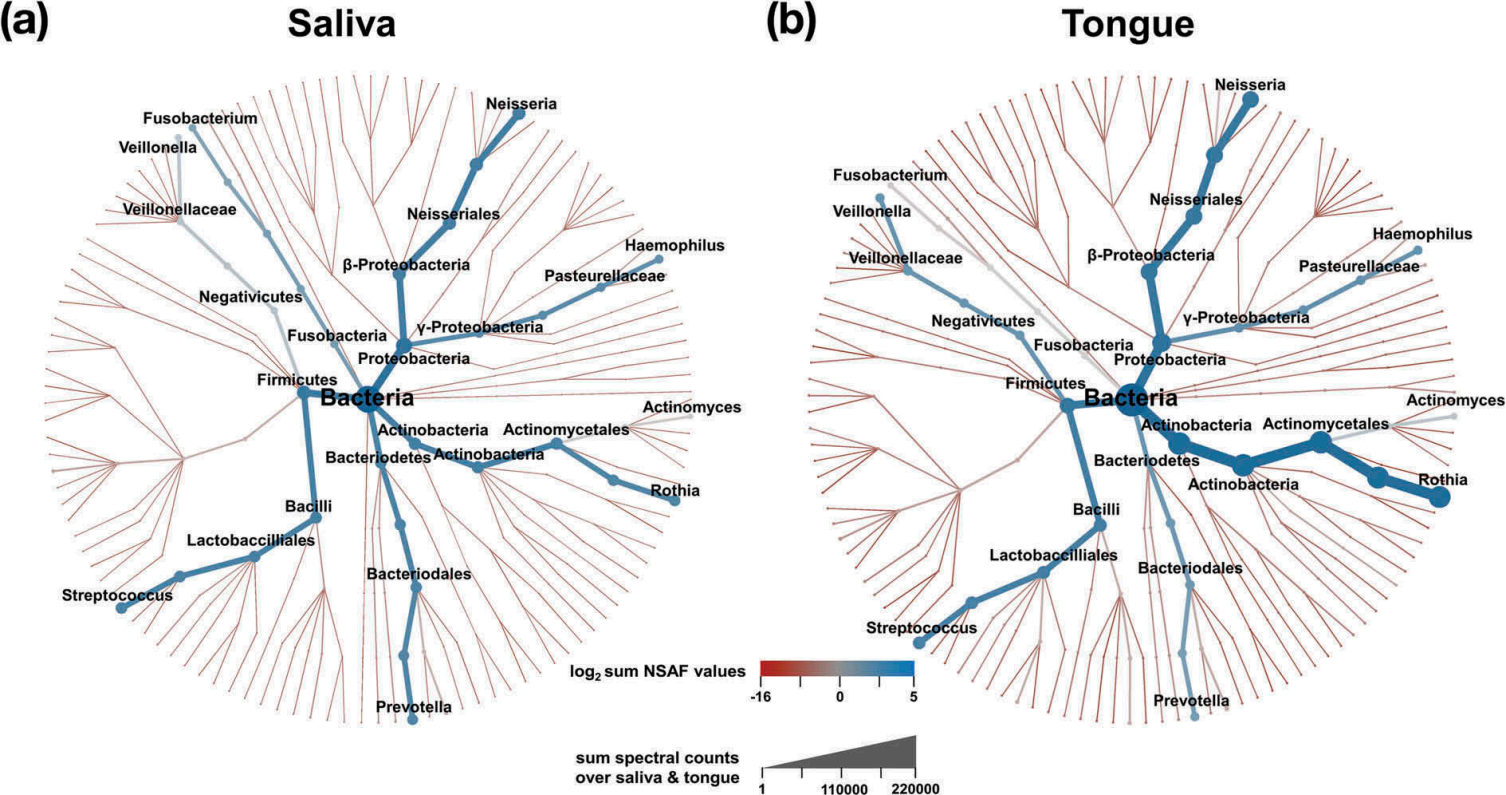
Heat trees of taxonomic composition of the healthy saliva (a) and tongue (b) microbiome. Coloration is defined by log_2_ sum normalized spectral abundance factors (NSAF-values) [60]. The number of spectral counts for each branch determines its thickness.

In total, we identified seven phyla (Supplemental Figure 3), of which *Actinobacteria, Bacteriodetes, Firmicutes, Fusobacteria*, and *Proteobacteria* were most common and have been detected in all subjects. A comparison of the two sample types revealed that *Proteobacteria* and *Firmicutes* appeared almost in an equal abundance, while *Actinobacteria* emerged as more abundant on the tongue. *Bacteriodetes* and *Fusobacteria* showed a contrary trend and were identified in smaller abundances on the tongue. The two other phyla *Spirochaetes* and *Synergistetes* contributed with only a small proportion to the bacterial community. Furthermore, we could identify *Chlamydiae*, but only for five subjects in saliva and therefore they were excluded from further analysis. At the genus level we could assign 93.9 % of all 3,969 bacterial metaproteins to 107 different genera and we found a high similarity between saliva and tongue with an overlap of 89 genera (83.0%). To gain insight into the distribution at the genus level, we created heat trees where summed relative abundances of spectral counts were plotted (Figure 3(a,b)). In general, we identified higher bacterial abundances on the tongue in comparison to saliva. For saliva and the tongue, we recognized a high bacterial diversity but only the seven genera *Rothia, Prevotella, Streptococcus, Veillonella, Fusobacterium, Neisseria*, and *Haemophilus* mainly determined the composition of both microbiomes. In summary, we could observe a great diversity in saliva and tongue dominated by seven phyla and genera, but we could only observe small and non-significant differences when the two sample types were compared to each other (Supplemental Table 3). Likely more subtle differences were masked by the large interindividual differences in the microbiomes observed in this and other studies.

**Figure 4.**
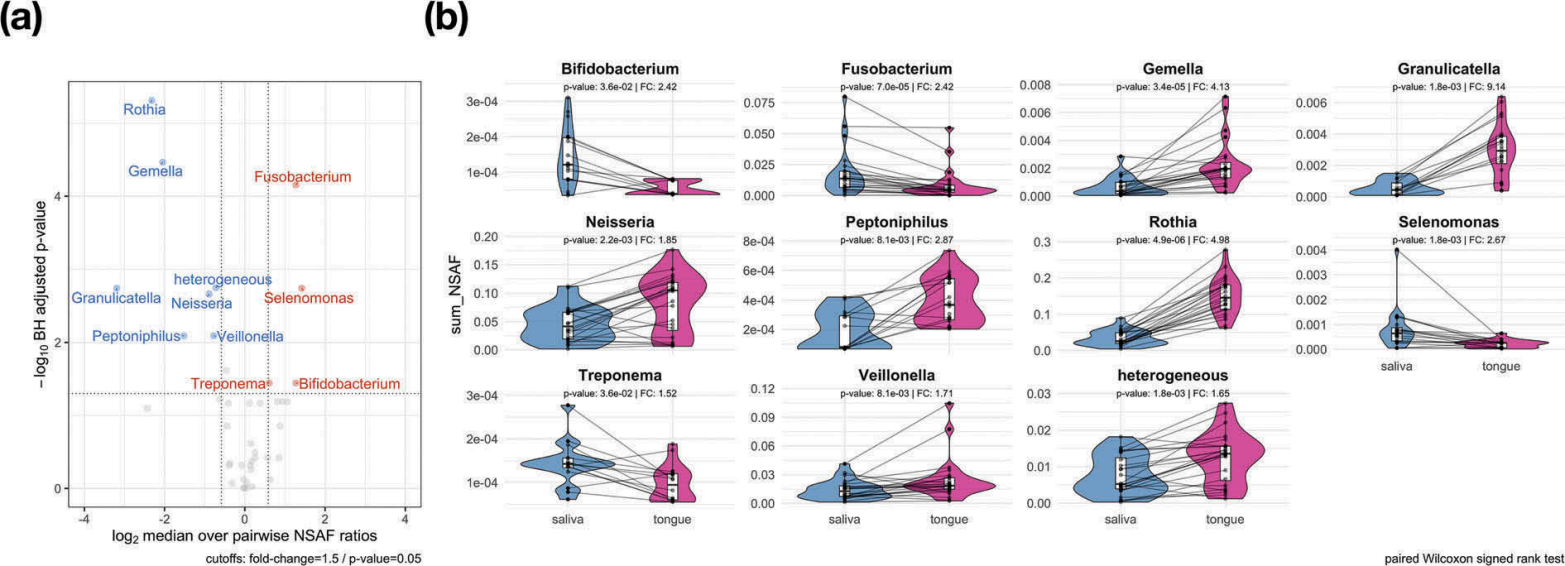
Significant taxonomic profile differences on the genus level between saliva and tongue are displayed in the volcano plot (a) by depicting the results of a two-paired Wilcoxon signed rank test. The comparison plots (b) show the sum of the NSAF values for those genera identified as significantly higher abundant in saliva or on the tongue. Metaproteins in the group ‘heterogeneous’ could not be assigned unambiguously to a genus.

To increase the sensitivity of our analyses, we performed a pairwise analysis (paired Wilcoxon signed rank test, p-value: < 0.05) at genus level. Figure 4(a,b) illustrates ten genera with significant differences. *Fusobacterium, Selenomonas, Bifidobacterium* and *Treponema* were found to be significantly increased in saliva compared to the tongue. The opposite was the case for *Rothia, Gemella, Granulicatella, Peptoniphilus, Veillonella* and *Neisseria*. Furthermore, we identified significant changes in the category ‘heterogeneous’, which cannot be further described, since an assignment on genus level was not feasible.

**Figure 5.**
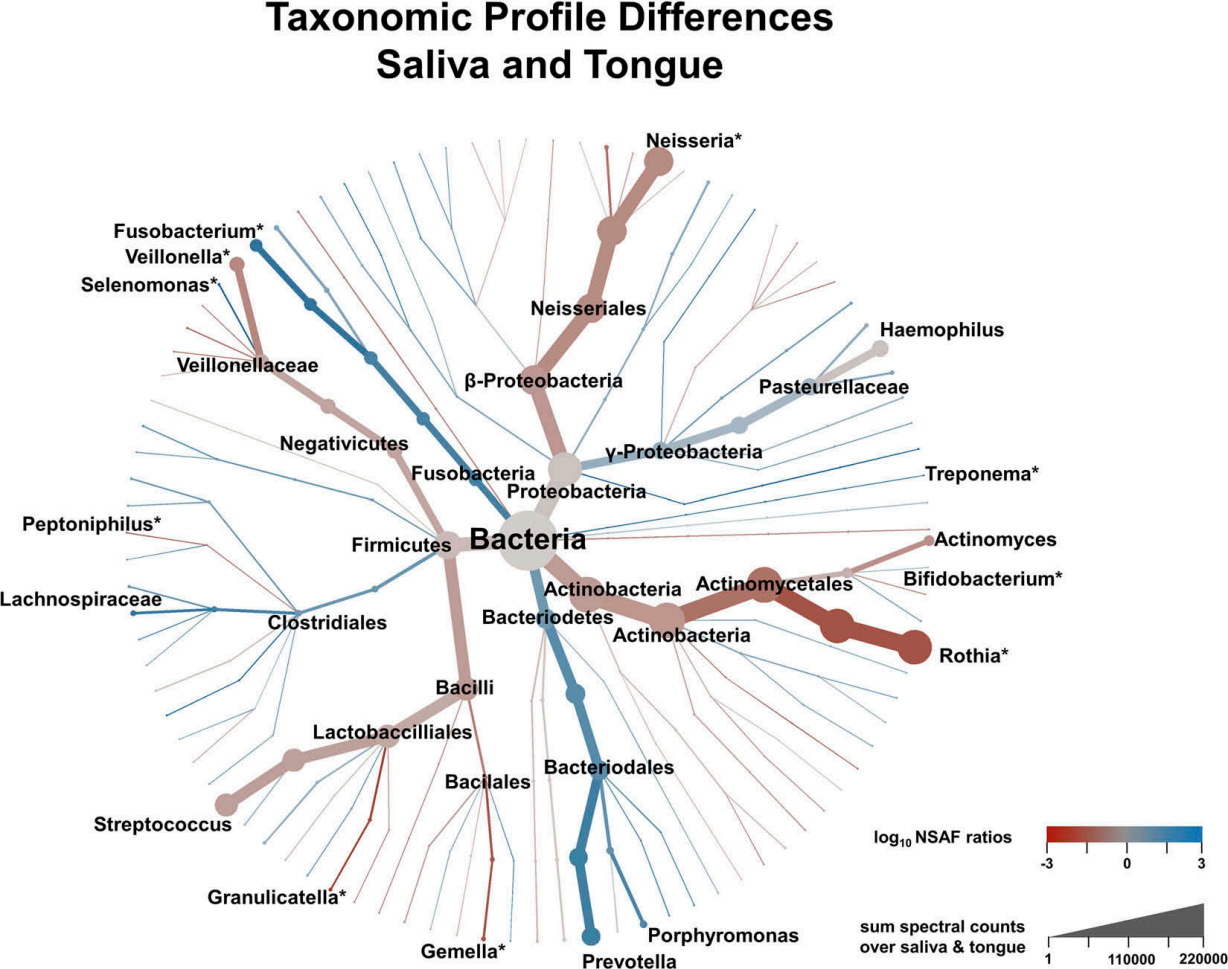
Illustration of taxonomic differences between saliva and tongue based on median over pairwise NSAF ratios (coloration) and the sum of spectral count (branch size). Genera marked with an * showed significant differences between both microbiomes according to a Wilcoxon signed rank test (Benjamini-Hochberg corrected p-value < 0.05).

Figure 5 provides a more detailed picture of the differences in taxonomic profiles of saliva and tongue samples by representing the complete calculated ratios in a phylogenetic tree. In combination with the results of the Wilcoxon signed rank test, it highlights the differences regarding the taxonomic composition of the two microbiomes. Even though *Rothia, Veillonella, Fusobacterium* and *Neisseria* belonged to the dominant genera, they also showed great differences between saliva and tongue, which was not the case for the genera *Prevotella, Streptococcus, Haemophilus* and *Actinomyces*. Genera such as *Granulicatella, Gemella, Peptoniphilus* or *Bifidobacterium*, which do not dominate the two microbiomes and would thus not to be noticed at first glimpse, also showed relevant and significant differences.

We also analyzed, whether we could identify any gender differences in the microbiome composition, but our results did not indicate any significant and specific microbiomes for males or females (paired t-test, p-value: < 0.05; fold-change > 2).

### Functional profiling of bacterial metaproteins

**Figure 6.**
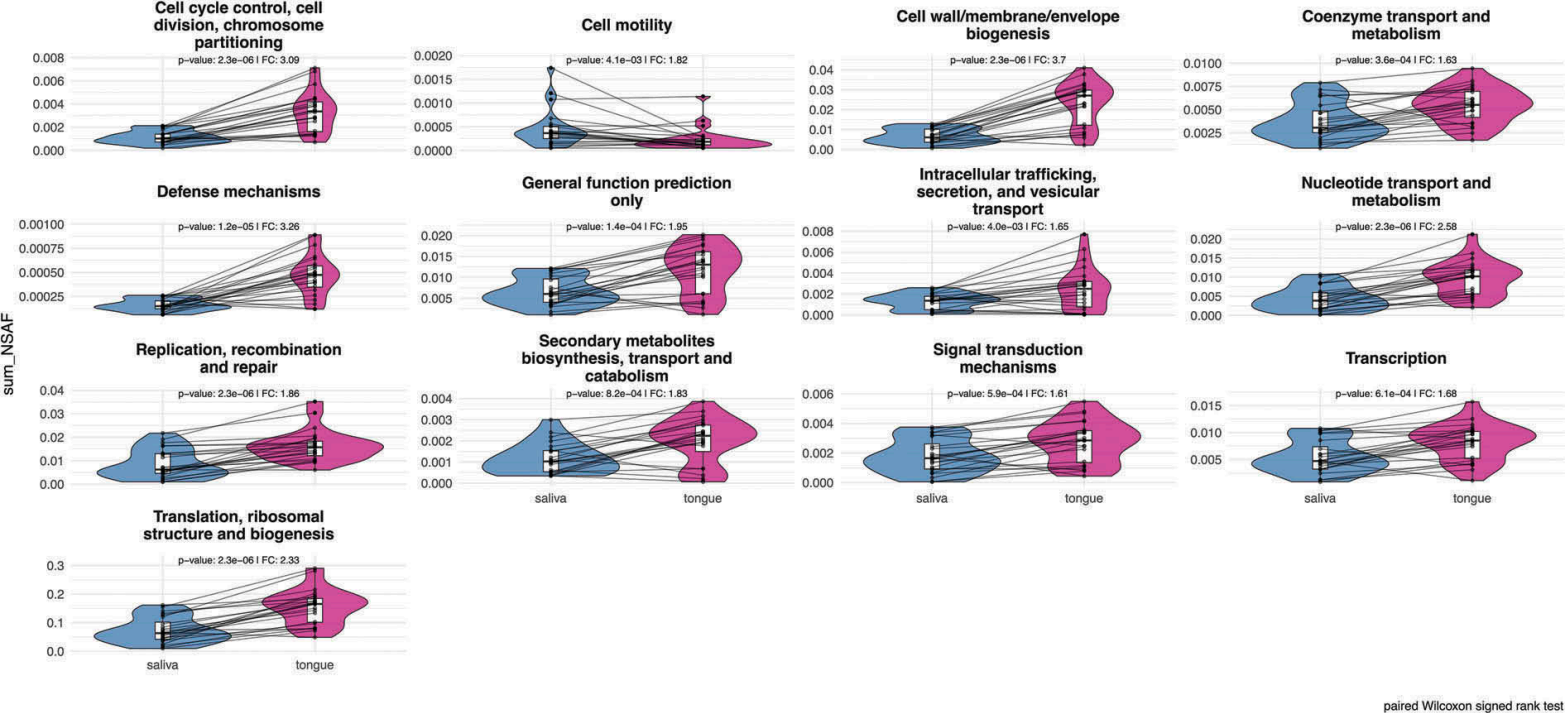
Comparison plots show the different relative abundances of bacterial metaprotein functions with significant differences, which were determined by a two-sided pairwise Wilcoxon signed rank test (p-value < 0.05) with a fold change of > 1.5. The calculated p-value has been corrected according to the Benjamini-Hochberg method.

Metaproteome analyses enable simultaneous assessment of expressed human and bacterial metabolic pathways. From a global point of view, we covered 18 biological processes based on the COG classification [57] for bacterial metaproteins (Supplemental Figure 4) in saliva and on the tongue. The most common functions were translation, energy production, carbohydrate metabolism and amino acid metabolism (Supplemental Table 4). Again, there was a strong similarity between saliva and tongue regarding functional composition at this global perspective. As expected, due to their high abundance the functional profile was dominated by metaproteins involved in translation with an averaged portion of 40.2% and 29.8% for the tongue and saliva, respectively. Processes like cell cycle, secondary metabolites, intracellular transport, signal transduction, defense mechanism and cell motility made up less than 1%, but again in a pairwise analysis (paired Wilcoxon signed rank test, p-value: < 0.05) all these functions with the exception of cell motility were found significantly increased on the tongue compared to saliva (Figure 6). A similar conclusion could be reached for metaproteins that are involved in bacterial cell wall biogenesis, coenzyme and nucleotide metabolism as well as in replication, transcription and translation processes. Thus, only metaproteins of cell motility displayed increased levels in saliva. We were not able to determine the exact functions of all metaproteins, and thus the remining proteins were summarized in the category ‘general function prediction only’, which was also significantly different in saliva and tongue.

### Functional profiling of human proteins

**Figure 7.**
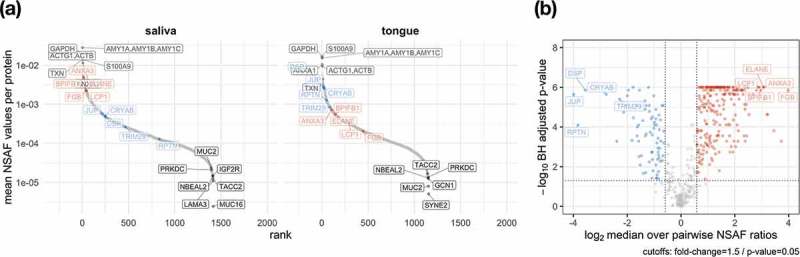
The coverage of the dynamic range of human proteins is shown by plotting the mean relative abundance for saliva and tongue (a). The human proteins are named according to their gene names and show for saliva and tongue a selection of proteins with highest and lowest abundances (grey). Data points in red and blue display proteins with a fold change > 1.5 and a p-value < 0.5 (paired Wilcoxon signed rank test) comparing saliva and tongue (a). Proteins with the largest changes are highlighted with their gene names (A/B).

As already shown in Figure 2(a,c), there was a great overlap between human proteins in saliva and on the tongue, which remains observable by ranking proteins based on their relative abundance and considering the top highest and lowest abundant proteins (Figure 7(a)). Some of the highest proteins identified in saliva and on the tongue were alpha-amylase (AMY1), which catalyses the digestion of starch and glycogen [65], glyceraldehyde-3-phosphate dehydrogenase (GAPDH) for the reversible oxidative phosphorylation of glyceraldehyde-3-phosphate [66] or the phospholipase A_2_ inhibiting protein annexin A1 (ANXA1) [67]. However, proteins such as the laminin subunit alpha 3 (LAMA3) belonging to the laminin family, the glycoprotein mucin 2 (MUC2) [68] or the F-actin-binding species repeat containing nuclear envelope protein 2 (SYNE2) [69] could only be found at low abundance.

**Figure 8.**
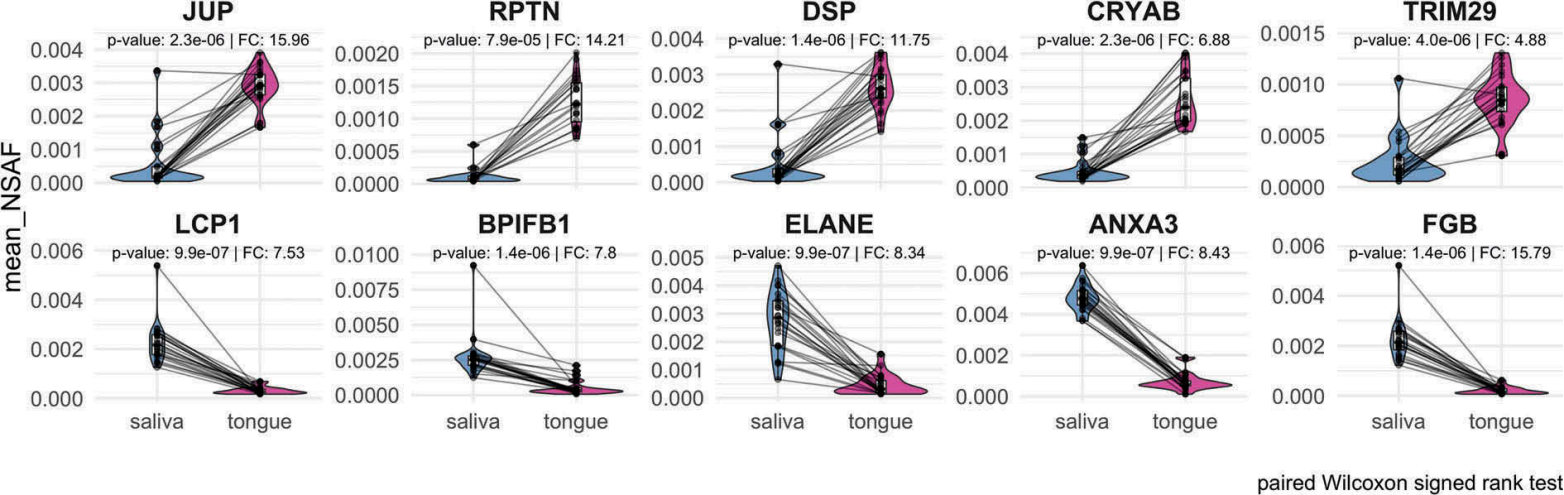
Representation of the top five proteins with the highest increase or decrease regarding to their relative abundance in saliva or on the tongue based on paired Wilcoxon signed rank test (p-value < 0.05).

Pairwise analysis of human proteins revealed that 75 proteins occur in saliva in significantly lower abundance than on the tongue, while 232 proteins were significantly higher in saliva compared to the tongue surface (Figure 7(b)). Many proteins with increased abundance in saliva (Figure 8) play a role in the innate or adaptive immune system (Lypmhocyte cytosolic protein 1 – LCP1 [70]; BPI fold containing family B member 1 – BPIFB1 [71]; Elastase – ELANE [72]; Annexin A3 – ANXA3 [67]). Proteins with a higher abundance on the tongue could be assigned to the cytoskeleton (Figure 8), e.g. Junction plakoglobin (JUP) and Desmoplakin (DSP), playing a role in the regulation of innate immunity (Tripartite motif containing 29 – TRIM29) [73] or prevent possible irreversible protein aggregations as chaperones (Crystallin alpha B – CRYAB).

## Discussion

The primary goal of this study was to describe and compare the human saliva and tongue microbiome of healthy young individuals. Initially, we wanted to explore whether we were able to achieve comparable results in protein identification with the pipeline described in this report compared to other metaproteome studies [37–40]. For the tongue analyses we identified 4,644 proteins of which more than 70% originated from bacterial species. To the best of our knowledge, this is the first study providing metaproteome data for the tongue. Regarding salivary proteins, we profiled slightly less human proteins compared to Grassl et al. and BelstrØm et al. [39,40]. At the same time, the number of bacterial proteins covered was slightly higher in the current study. Possible reasons include the use of different or modified protocols for sample collection, preparation and measurement [37–40]. The same applies to the different data analysis strategies, which have an impact on the peptide-protein assignment [44,45,74,75], especially for bacterial proteins due to the high number of different taxa [76], which results in many shared peptides on the protein level [55]. Furthermore, the number of subjects and particularly the cohorts differed. Whereas former studies included diseased subjects [38,40], in whom large interindividual differences must be expected, especially in the case of bacteria [22,24], our study was confined to young healthy participants to define baseline-microbiomes of the healthy population.

For future studies, we also wanted to clarify whether it is necessary to analyze technical replicates to obtain reliable metaproteome results and whether the related significant increase in measurement time is associated with a relevant increase in protein identifications. It became apparent that in saliva with a technical variance of 16%, we achieved similar results as previous (meta-) proteome studies [36,77]. Additionally, measuring three replicates, we achieved an increase in protein identification of around 28.2%. However, considering the good technical reproducibility of the data and the threefold increase in measurement time, we do not consider replicate measurements to be the preferred solution. Rather, we propose to cover the diversity of the metaproteome and thus of the microbiome by measuring more samples from different individuals, since our data and other microbiome studies point to large interindividual differences [5,8,9,24,27].

As another aspect of this study, we investigated the relative abundance of human and bacterial proteins, where we expected a higher proportion of human proteins in saliva than on tongue. We can confirm these expectations with our data for several reasons. Bacteria in saliva are planktonic whereas on the tongue bacteria are likely organized in a biofilm [38]. In addition, saliva consists to 99% of water [78], which may lead to a dilution of the bacteria. Furthermore, human proteins are two orders of magnitude more abundant than those of bacteria [39], which leads to the suppression of less abundant proteins during the measurement [37]. In particular, alpha-amylase (AMY1A), or S100 calcium binding protein A9 (S100A9) have to be mentioned, both displaying high abundances in our saliva and tongue data, an observation also made for saliva before [79]. We also detected alpha-amylase (AMY1A) [65], glyceraldehyde-3-phosphate dehydrogenase (GAPDH) [66], annexin A1 (ANXA1) [67] and 62% of all saliva human proteins on the tongue, which indicate that the human part of the tongue surface proteome is, as expected, partially shaped by the surrounding saliva [5,9].

The assignment of proteins to specific bacterial species is a challenge for metaproteomic studies due to protein homologies [55,74]. We could also confirm the observations made by BelstrØm et al. [40] and Rudney et al. [38], which showed that the assignment of proteins to certain taxa decreases when approaching the species level.

To the best of our knowledge, this is not only the first study providing a metaproteomic description for the tongue but also its comparison with saliva. Saliva and tongue microbiome both revealed a high diversity, dominated by the seven genera *Rothia, Prevotella, Streptococcus, Veillonella, Fusobacterium, Neisseria*, and *Haemophilus*. For saliva we confirmed the results of previous metaproteome studies at the phylum and genus level [37–40]. Thus, *Actinobacteria, Proteobacteria, Firmicutes, Bacteriodetes* and *Fusobacteria* were also the five most abundant phyla [80]. The observed high diversity is probably caused by a large interindividual microbial variation especially at the lower taxonomic levels [22,81]. Additionally, our data indicated that saliva and tongue microbiomes display a strong taxonomic similarity, which is in agreement with a comparative 16S RNA study of saliva and tongue microbiomes conducted by Hall et al. [24] as well as a study from Papaioannou et al. [25]. At the phylum level, we initially found no significant differences between saliva and tongue in a general comparison of both sample types. Increasing sensitivity by pairwise analysis of the samples from the same individuals, the impact of interindividual differences could be reduced and significant differences at genus level between both microbiomes were revealed [80,82]. Our data indicate, that even genera, which do not dominate the microbiome do clearly contribute to the differences between the two microbiomes.

Most genera were also present in higher abundances on the tongue, which could provide a further hint that the tongue might be a reservoir contributing to the composition of the saliva microbiome [24]. This may suggest that more attention might need to be paid to the tongue in oral hygiene, since pathogenic bacteria seem to be present even in a healthy microbiome [11,38] and could be distributed from the tongue throughout the oral cavity by saliva [24].

Although metaproteome studies are not as sensitive for the determination of bacterial diversity as metagenome studies, they provide the decisive advantage of analyzing expressed metabolic pathways and thus metabolic activity [35].

Analysis of protein functions demonstrated besides taxonomic also functional similarity with relevant characteristics between saliva and tongue. Our findings are in line with previous observations that despite an interindividual diversity between different habitats a functional conservation exists [83]. At least 30% of the identified proteins play a role in translation and especially different ribosomal proteins have been found, which supports the hypothesis that these proteins are essential for (growing) microbes [84] and are therefore highly conserved and abundant in metaproteomic samples [38]. Bacteria in saliva are in a planktonic state, which might be an explanation that we identified a significantly increased number of metaproteins especially with functions in cell motility [38]. On the other hand, the tongue microbiome exists as a biofilm with significantly different environmental conditions [2]. Biofilms are continuously exposed to the human immune system, which might explain the increased abundance of defense mechanisms metaproteins [85]. The increased occurrence of metaprotein functions like signal transduction and secondary metabolites may suggest increased intra- and inter-bacterial communication [86]. Reasons for this could include competitive or mutualistic behavior [87]. Metaproteins with functions in replication, transcription and translation might indicate a still growing biofilm [88].

For some of the metaproteins it was not possible to determine their functions. The same applied to the taxonomic classification of metaproteins, which were classified as ‘heterogeneous’. Here, currently existing databases as well as analysis tools reached their limits. In this case, future metaproteome analyses will benefit enormously from improved databases and analysis tools, which will enable a better assignment of metaproteins on a taxonomic as well as functional level [37,89].

Besides the digestion of glycogen by alpha-amylase, another important function of saliva is the maintenance of the balance of the microbiome and the defense against pathogens by the immune system [90]. This could be an explanation for the significantly increased number of human proteins, whose functions were mainly involved in the immune response system.

The tongue is a muscular organ with a keratinized stratified squamous epithelium and mostly cytoskeletal proteins or the repetin (RPTN) involved in the cornified cell envelope formation have been identified [91], which we attribute to the fact that the sample material was scraped off directly from the tongue, whereas saliva is a mixture from the salivary glands [92].

Limitations of our study include the unequal distribution of male and female subjects as well as the rather small number of 24 subjects. Therefore, like Grassl et al. [35] we could not detect sex specific differences in the microbiome. Nevertheless, we consider the question about the *microgenderome* [93] to be important and worth studying [94]. So far, besides anti-microbial effects of saliva [79], also significant differences in the salivary microbiome of male and female children [95], possibly due to the endocrine system, have been described. Women have about twice the chance of getting caries than men [78] and thus Lukacs and Largaespada hypothesized that possible reasons could be factors like a reduced salivary flow rate and hormone fluctuations [78], which likely influence the microbiome. Gender differences in the microbiome are also further supported by current studies of the gut microbiome, which were able to detect differences between men and women [96–98].

For this ‘proof of principle’ study a selected cohort of dentistry students with a defined small age range was selected. Future metaproteome studies addressing the healthy oral microbiome in a larger cohort should provide a better demographic [99] and geographical diversity [100,101]. In addition, it must be clarified under which criteria a microbiome can be considered as ‘healthy’ [28]. This definition is not a trivial task as previous discussions have shown [102–104]. In addition to the recording of clinical parameters, the personal oral hygiene of the subjects [105], their diet [106], genetic background [107], socio-economic status [108] as well as other aspects must be considered, which will increase the effort and complexity of a study significantly. It must e.g. also be clarified, which influence the circadian change of the flow rate of saliva has on the time of sample collection [109,110], even if previous studies have shown temporal stability of the oral microbiome over a longer period [5,111,112].

Therefore, we conclude that several basic issues still need to be addressed in future studies of oral microbiomes. Nevertheless, providing many different parameters for a cohort increases the quality of a study, which is particularly important for clinical studies that want to distinguish between healthy and diseased microbiomes [23,29].

## Conclusions

Our metaproteome study aimed to provide a detailed insight into the taxonomic composition and functional diversity of saliva and tongue in 24 healthy young adult volunteers. This is the first study, which described the healthy tongue microbiome of young subjects and compared it to saliva based on metaproteome data. Therefore, we have developed a strategy to evaluate large metaproteome data sets by combining TPP and Prophane. Essentially, we found a high bacterial diversity for saliva and tongue, which was mainly determined by seven genera. Globally, we identified high taxonomic similarity and functional consistency between both microbiomes, although we must emphasize that interindividual differences strongly influence the taxonomic composition. However, using comparison of paired samples from the same individuals, we were also able to show decisive functional differences of bacterial metaproteins between the biofluid saliva and the tongue biofilm. The good agreement of our results with those of already performed metagenome and metaproteome studies demonstrated that our workflow can provide consistent metaproteomic results.

To ensure an even better description of the different human microbiomes, future studies should focus on multi-OMICs approaches. Furthermore, the size of cohorts needs to be increased to enable a more precise identification of interindividual differences, which should allow a more accurate description of the microbial profile of a healthy microbiome and the distinctive features from dysbiotic states in pathological situations.
