# Supplementary material for: Metaproteomics analysis of microbial diversity of human saliva and tongue dorsum in young healthy individuals
Source: J Oral Microbiol. 2019 Aug 26;11(1):1654786. doi: 10.1080/20002297.2019.1654786 (PMC6720020; doi:10.1080/20002297.2019.1654786)
Supplement: Supplemental Material [file ZJOM_A_1654786_SM9835.zip › ZJOM_A_1654786_Supplementary/03_sal_tong_healthy_microbiome_supplemental_JoOM.docx]

**Supplemental Table 1:** Measurement parameters for mass spectrometry

| ***reversed phase liquid chromatography (RPLC)*** | |
| --- | --- |
| instrument | Ultimate 3000 RSLC (Thermo Scientific) |
| trap column | 75 μm inner diameter, packed with 3 μm C18 particles (Acclaim PepMap100, Thermo Scientific) |
| analytical column | Accucore 150-C18, (Thermo Fisher Scientific)  25 cm x 75 μm, 2,6 μm C18 particles, 150 Å pore size |
| buffer system | binary buffer system consisting of 0.1% acetic acid water (buffer A) and 100% ACN in 0.1% acetic acid (buffer B) |
| flow rate | 300 nl/min |
| gradient | linear gradient of buffer B from 2% up to 25% |
| gradient duration | 120 min |
| column oven temperature | 40°C |
| ***mass spectrometry (MS)*** | |
| instrument | Q Exactive plus mass spectrometer (Thermo Scientific) |
| operation mode | data-dependent |
| ***Full MS*** |  |
| MS scan resolution | 70,000 |
| AGC target | 3e6 |
| maximum ion injection time for the MS scan | 120 ms |
| scan range | 300 to 1650 m/z |
| spectra data type | profile |
| ***dd-MS2*** |  |
| resolution | 17,500 |
| MS/MS AGC target | 2e5 |
| maximum ion injection time for the MS/MS scans | 120 ms |
| spectra data type | centroid |
| selection for MS/MS | 10 most abundant isotope patterns with charge ≥2 from the survey scan |
| isolation window | 3 *m/z* |
| fixed first mass | 100 m/z |
| dissociation mode | higher energy collisional dissociation (HCD) |
| normalized collision energy | 27.5% |
| dynamic exclusion | 30 s |
| charge exclusion | 1,>6 |

**Supplemental Table 2:** Overview of spectra, peptides and proteins identified

| ***Sample*** | ***Number of Spectra***  **mFDR ≤ 0.06 %** | ***Number of Peptides***  **pepFDR ≤ 1.43 %** | ***Number of Proteins***  ***protFDR ≤ 5.00 %*** | | |
| --- | --- | --- | --- | --- | --- |
|  |  |  | ***Human*** | ***Bacteria*** | ***Total*** |
| Saliva | 990,540 | 31,386 | 1,647 | 2,633 | 4,280 |
| Tongue | 942,850 | 31,215 | 1,337 | 3,307 | 4,644 |
| Total | 1,933,390 | 62,601 | - | - | - |

**Supplemental Table 3:** Calculated p-values based on a Kruskal-Wallis test (p-value < 0.05) for seven identified phyla in saliva and tongue samples.

| ***Phylum*** | ***p-value*** |
| --- | --- |
| *Actinobacteria* | 0.24 |
| *Bacteriodetes* | 0.52 |
| *Firmicutes* | 0.36 |
| *Fusobacteria* | 0.29 |
| *Proteobacteria* | 0.32 |
| *Spirochaetes* | 0.49 |
| *Synergistetes* | 0.48 |

**Supplemental Table 4:** Averaged functional protein abundances for saliva and tongue based on COG classification.

| ***COG - subrole*** | ***Saliva [%]*** | ***Tongue [%]*** |
| --- | --- | --- |
| Amino acid metabolism | 9.71 | 8.02 |
| Carbohydrate metabolism | 14.37 | 10.19 |
| Cell cycle | 0.44 | 0.82 |
| Cell motility | 0.16 | 0.04 |
| Cell envelope | 2.70 | 6.04 |
| Coenzyme metabolism | 1.37 | 1.27 |
| Defense mechanism | 0.04 | 0.08 |
| Energy production | 14.12 | 11.22 |
| Inorganic ion metabolism | 4.68 | 3.77 |
| Intracellular transport | 0.52 | 0.58 |
| Lipid metabolism | 4.44 | 2.27 |
| Nucleotide metabolism | 1.46 | 2.26 |
| PTMs | 9.55 | 5.82 |
| Replication | 3.42 | 4.34 |
| Secondary metabolites | 0.43 | 0.48 |
| Signal transduction | 0.67 | 0.60 |
| Transcription | 2.14 | 1.97 |
| Translation, ribosomal structure | 29.79 | 40.25 |
