# Supplementary material for: Metaproteomics analysis of microbial diversity of human saliva and tongue dorsum in young healthy individuals
Source: J Oral Microbiol. 2019 Aug 26;11(1):1654786. doi: 10.1080/20002297.2019.1654786 (PMC6720020; doi:10.1080/20002297.2019.1654786)
Supplement: Supplemental Material [file ZJOM_A_1654786_SM9835.zip › ZJOM_A_1654786_Supplementary/supplementary figure caption.docx]

**Supplemental Figure 1.** Principal component analysis of log_2_ transformed MS/MS data of saliva samples revealing a greater interindividual than technical variance.

**Supplemental Figure 2.** Principal component analysis of log2 transformed MS/MS data of tongue samples revealing a greater interindividual than technical variance.

**Supplemental Figure 3.** Boxplots of the taxonomic composition of the saliva and tongue microbiome at the phylum level based on log_2_ normalized spectral abundance factors (NSAF-values) (61). No significant differences could be determined between saliva and tongue using a Kruskal-Wallis test (p-value ≤ 0.05).

**Supplemental Figure 4.** Bacterial metaprotein relative abundances (NSAF-values) of the healthy saliva and tongue microbiome based on the Cluster of Orthologous groups (COG) classification system.
