# Supplementary figures and images for: Metaproteomics analysis of microbial diversity of human saliva and tongue dorsum in young healthy individuals
Source: J Oral Microbiol. 2019 Aug 26;11(1):1654786. doi: 10.1080/20002297.2019.1654786 (PMC6720020; doi:10.1080/20002297.2019.1654786)

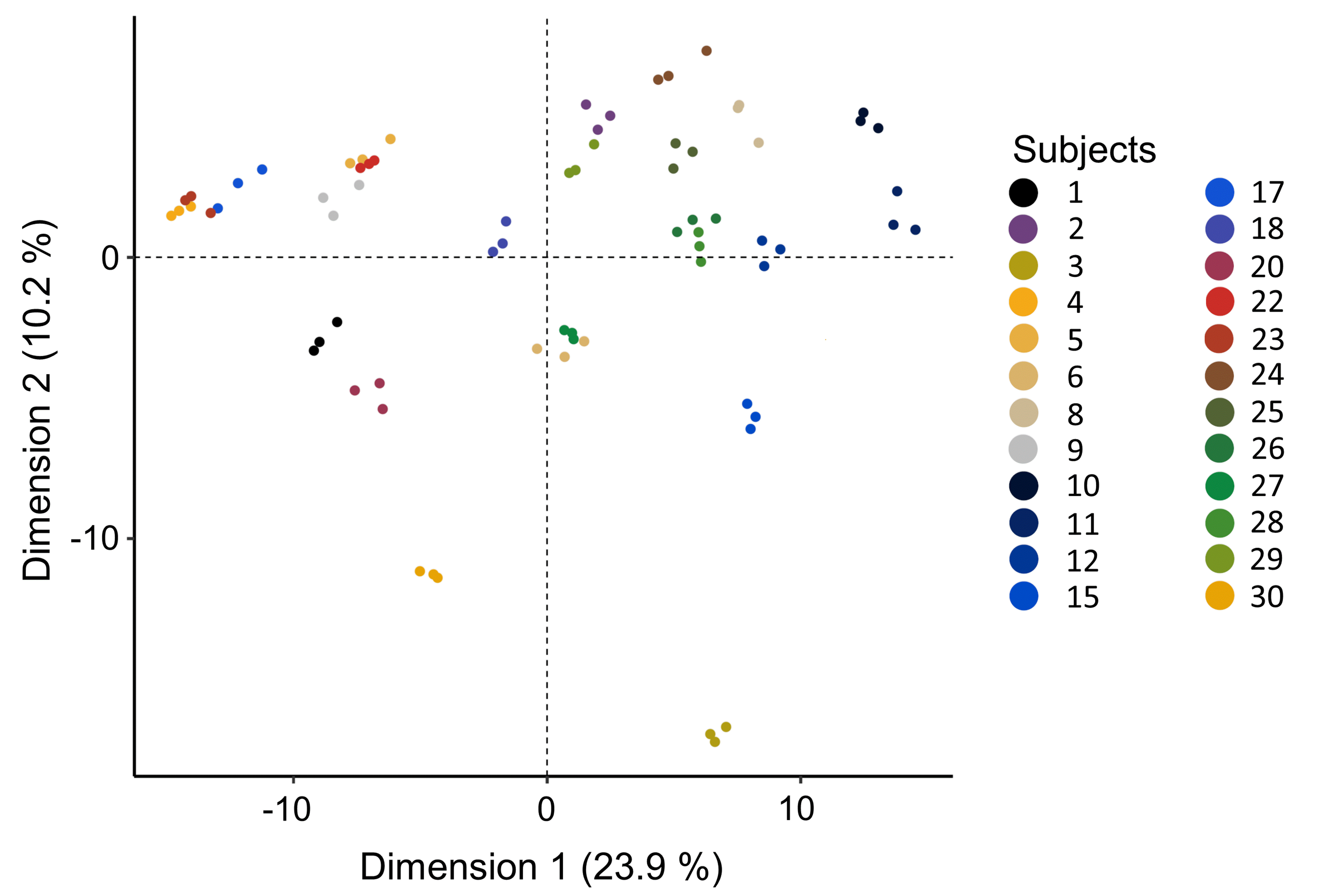

Supplement: Supplemental Material [file ZJOM_A_1654786_SM9835.zip › ZJOM_A_1654786_Supplementary/Supplemental_Figure_1_sal_tong_healthy_microbiome_JoOM.GIF]

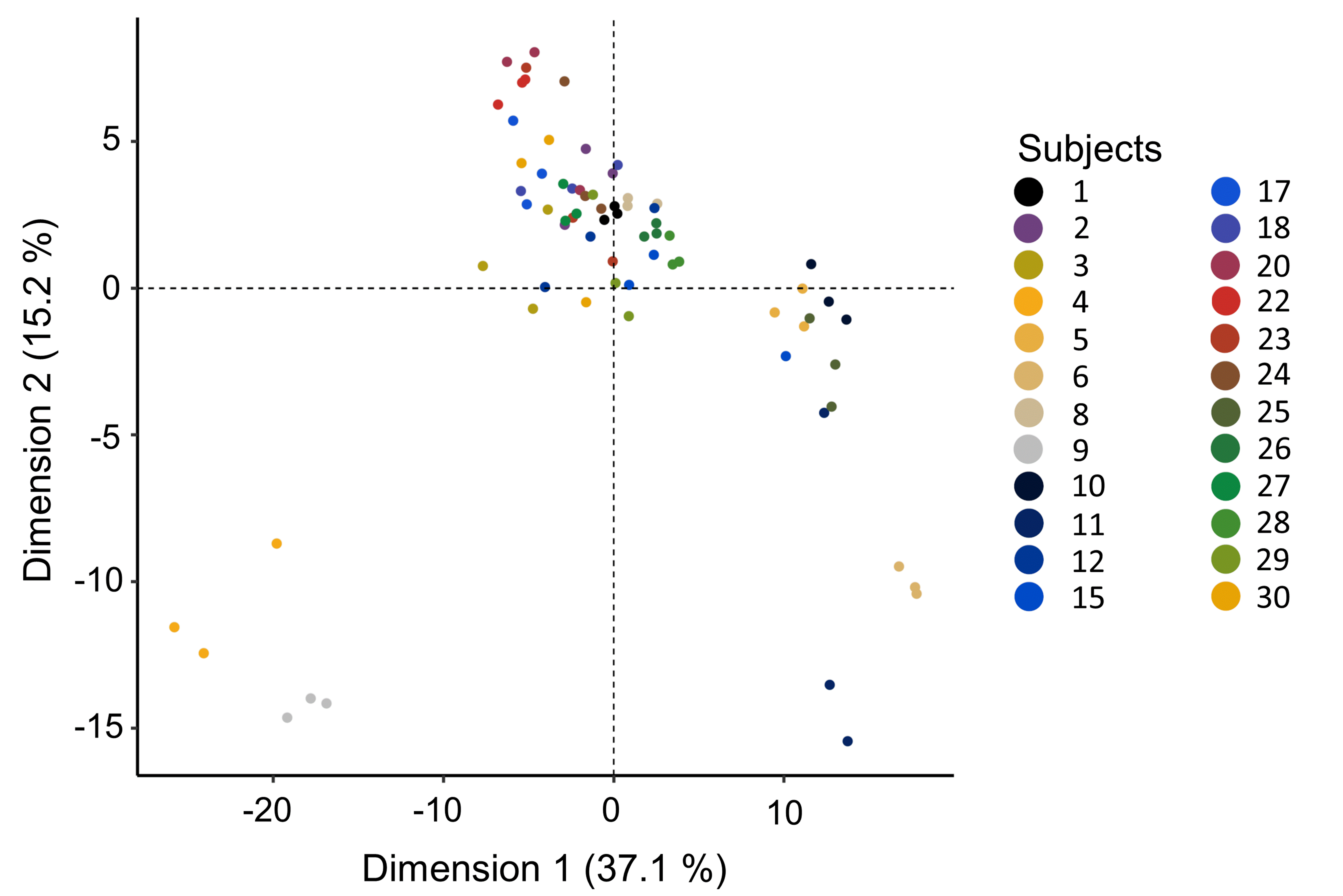

Supplement: Supplemental Material [file ZJOM_A_1654786_SM9835.zip › ZJOM_A_1654786_Supplementary/Supplemental_Figure_2_sal_tong_healthy_microbiome_JoOM.GIF]

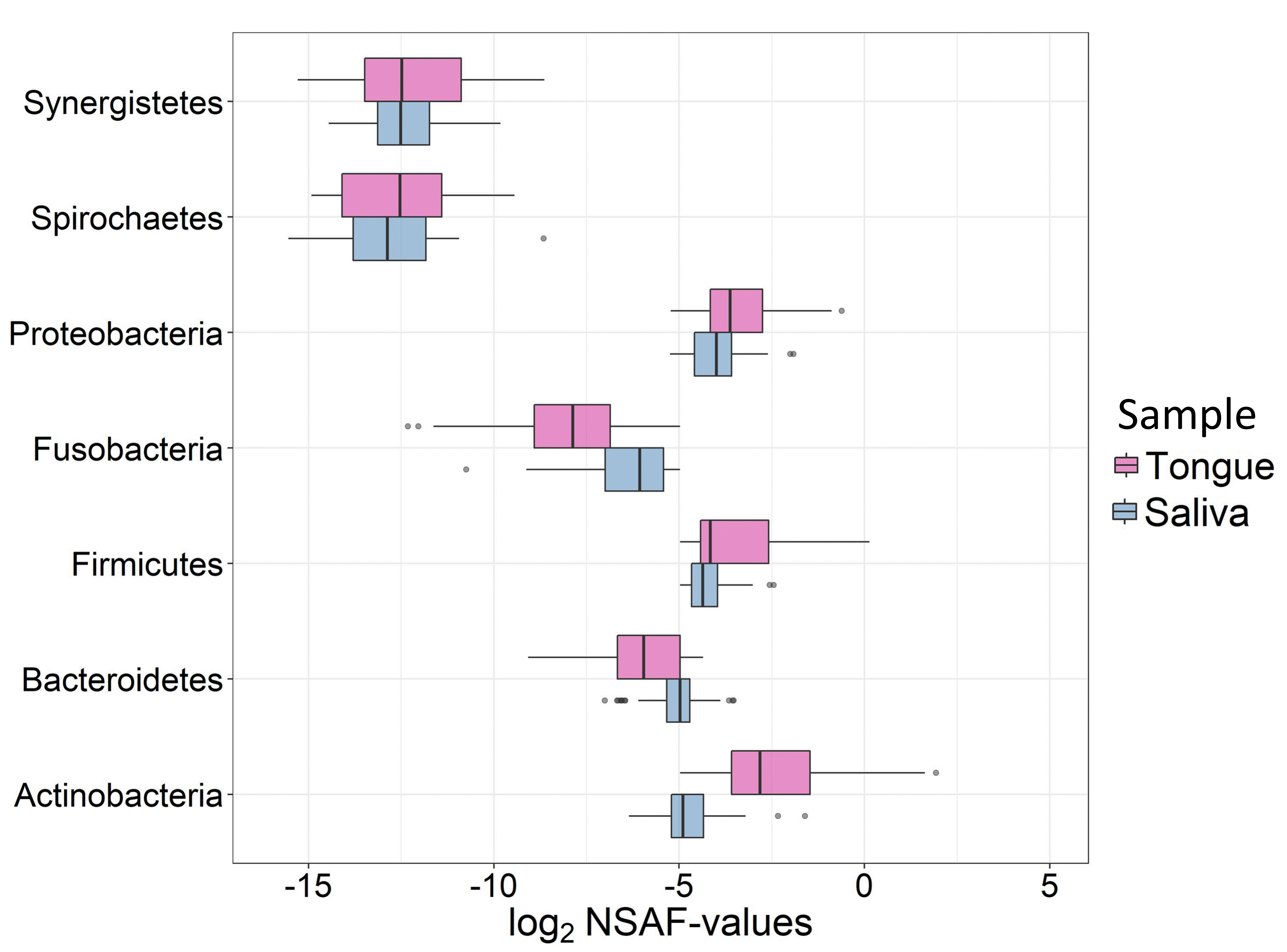

Supplement: Supplemental Material [file ZJOM_A_1654786_SM9835.zip › ZJOM_A_1654786_Supplementary/Supplemental_Figure_3_sal_tong_healthy_microbiome_JoOM.GIF]

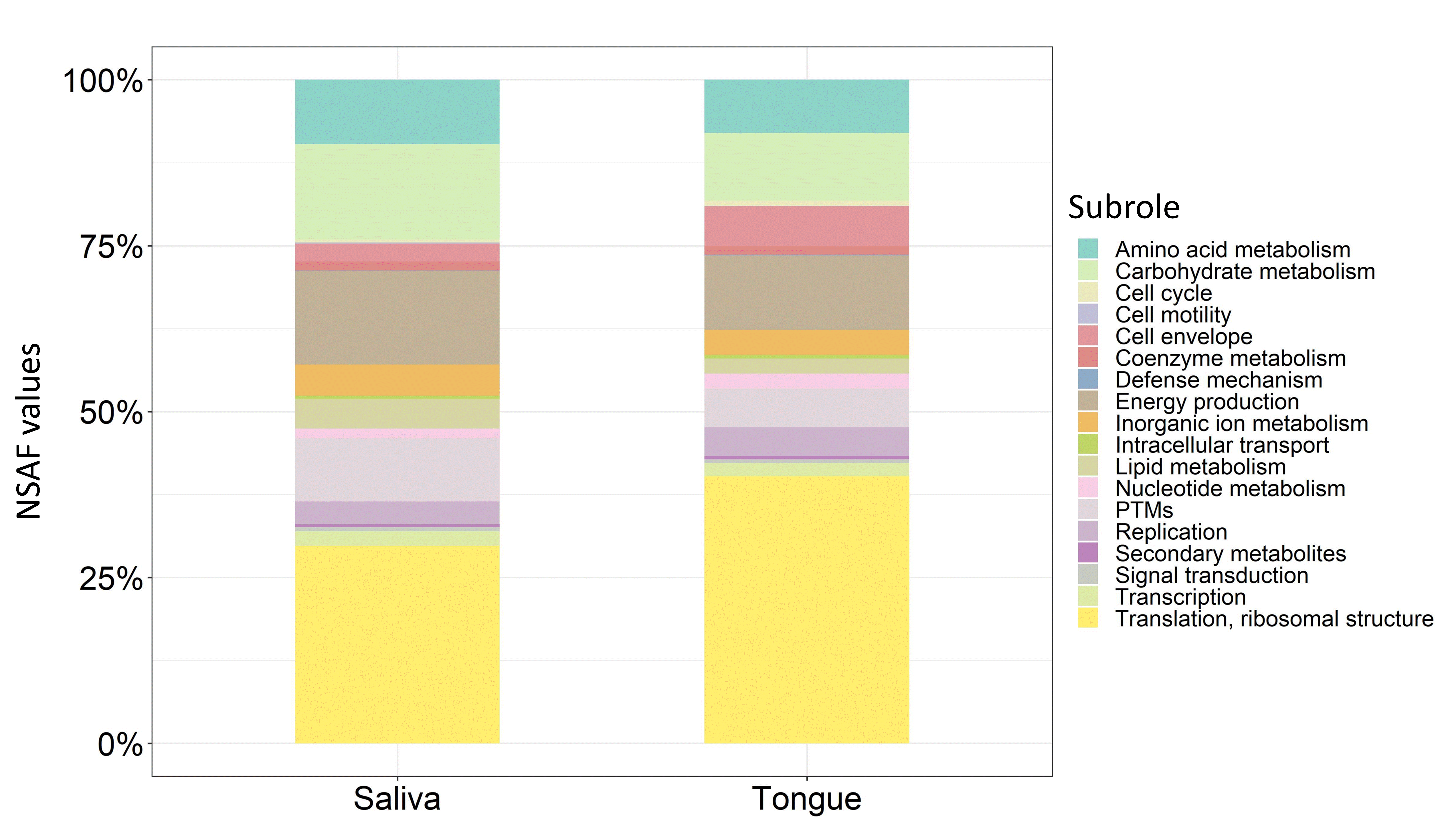

Supplement: Supplemental Material [file ZJOM_A_1654786_SM9835.zip › ZJOM_A_1654786_Supplementary/Supplemental_Figure_4_sal_tong_healthy_microbiome_JoOM.GIF]
